# Identification of a Torque Teno Mini Virus (TTMV) in Hodgkin’s Lymphoma Patients

**DOI:** 10.3389/fmicb.2018.01680

**Published:** 2018-07-26

**Authors:** Shaokun Pan, Tian Yu, Yanchun Wang, Renquan Lu, Huijie Wang, Youhua Xie, Xiping Feng

**Affiliations:** ^1^Department of Preventive Dentistry, Shanghai Ninth People’s Hospital, College of Stomatology, Shanghai Jiao Tong University School of Medicine, Shanghai, China; ^2^National Clinical Research Center for Oral Diseases, Shanghai Key Laboratory of Stomatology & Shanghai Research Institute of Stomatology, Shanghai, China; ^3^Key Laboratory of Medical Molecular Virology (MOH/MOE), Shanghai Medical College, Fudan University, Shanghai, China; ^4^Department of Oncology, Shanghai Medical College, Fudan University, Shanghai, China; ^5^Department of Medical Oncology, Shanghai Cancer Center, Fudan University, Shanghai, China

**Keywords:** lymphoma, Hodgkin’s lymphoma, anellovirus, torque teno mini virus (TTMV), viral metagenomics

## Abstract

At least 12% of human cancers are caused by virus infection. To understand whether other viruses are associated with human cancers, a viral metagenomics approach was used to analyze the composition of the viral communities of the serum of the patients with Hodgkin’s lymphoma (HL) and non-Hodgkin lymphoma. In this report, a human anellovirus TTMV named TTMV-SH was discovered from three patients with HL. The complete genome of TTMV-SH is 2812nt in length. Phylogenetic analysis based on ORF1 indicated that TTMV-SH of the 11 isolates cluster with TTMV strain TLMV-CBD231 sharing only 60.3–62% sequence similarity, and the sequences divergence is 41.5–43.1%, which indicates that TTMV-SH is a novel species. The TTMV-SH prevalence in HL group, especially in nodular sclerosing Hodgkin’s lymphomas (NSHL), was significantly higher than in the healthy group implicated that the TTMV-SH may be associated with HL, especially NSHL.

## Introduction

Virus infection cause at least 12% of human cancers (Mesri et al., 2014). Hepatitis B virus, Epstein-Barr virus, human papilloma virus, Kaposi’s sarcoma-associated herpesvirus, Merkel cell polyomavirus (DNA viruses), Human T lymphotrophic virus type 1 and Hepatitis C viruses (RNA viruses) have been shown to contribute to the development of human cancers (Lawson et al., 2016; Dylawerska et al., 2017; Gloghini et al., 2017; Ringelhan et al., 2017).

Lymphoma is a group of blood cancers that develop from lymphocytes. The two main types of lymphomas are Hodgkin’s lymphomas (HL) and the non-Hodgkin lymphomas (NHL) (Stewart and Wild, 2014). Risk factors for HL include infection with Epstein–Barr virus, a family history of the condition and having HIV/AIDS (Stewart and Wild, 2014; Gloghini et al., 2017; Linke-Serinsoz et al., 2017; Pozzato et al., 2017). While risk factors for common types of NHL include autoimmune diseases, HIV/AIDS, infection with human T-lymphotropic virus, infection with hepatitis C virus, immunosuppressant medications, and some pesticides (Poiesz et al., 1980; Stewart and Wild, 2014; Gloghini et al., 2017; Linke-Serinsoz et al., 2017; Pozzato et al., 2017).

Anelloviruses, family *Anelloviridae*, are non-enveloped, circular, single-stranded DNA viruses with genome range in size from 2.1 to 3.9 kb in length (Biagini, 2009; Spandole et al., 2015). Torque teno mini virus (TTMV) with the genome of 2.8–3.0 kb has one GC rich region and three ORFs, a large ORF1 with ∼70% genome length, ORF2 and ORF3.

According to International Committee on Taxonomy of Viruses (ICTV) classification, the family of *Anelloviridae* has been divided into 12 genera and 68 species. Alphatorquevirus (torque teno virus, TTV), Betatorquevirus (TTMV) and Gammatorquevirus (torque teno midi virus, TTMDV) are most studied anelloviruses (Fornai et al., 2001; Gergely et al., 2006; Biagini, 2009; Galmes et al., 2013; AbuOdeh et al., 2015; Spandole et al., 2015; Zhang et al., 2016, 2017). According to the current criteria, demarcating species and genera in the family *Anelloviridae* employs cut-off values for sequence divergence of full length ORF1: species > 35%, genera > 56% (Biagini, 2009).

In the present study, 271 human lymphoma and 40 healthy donor serum samples were investigated using a viral metagenomics approach. We found a novel species of TTMV virus in 3 out of 19 human HL serum samples, all three samples belong to nodular sclerosing Hodgkin’s lymphomas (NSHL), but not in 252 non-Hodgkin lymphoma and 40 healthy donor serum samples. It may be possible that the TTMV-SH may be associated with HL.

## Materials and Methods

### Sample Collection

A total of 19 serum samples of patients with HL (9 men and 10 women, aged 15–63 years), 252 serum samples of patients with Non-Hodgkin lymphoma (152 men and 100 women, aged 14–88 years) and 40 serum samples of healthy people without lymphoma (22 men and 18 women, aged 19–64 years) were collected from the Department of Clinical Laboratory, Shanghai Cancer Center for Next-generation sequencing, during 15 Sep 2015 to 30 July 2016 (**Table 1**). All the samples were stored at -80°C until use. This study was carried out in accordance with the recommendations of Shanghai Cancer Center medical ethics committee with written informed consent from all subjects. All subjects gave written informed consent in accordance with the Declaration of Helsinki. The protocol was approved by the Shanghai Cancer Center medical ethics committee (IRB No. 050432- 4-12128).

**Table 1:** Serum sample information.

	Male	Female	Average age	Age distribution
Hodgkin’s lymphoma	9	10	31.6	15–63
NSHL	3	7	27.9	15–41
MCCHL	6	2	37	15–63
NLPHL	0	1	29	29
Non-Hodgkin lymphoma	152	100	52.2	14–88
B-cell lymphoma	110	77	52.6	14–88
DLBCL	73	49	52	14–82
FL	16	13	51.7	14–80
MALToma	11	8	53	18–88
MCL	5	3	68.4	59–77
Burkitt lymphoma	2	0	31	14–48
Other B-cell lymphoma	3	4	54.2	17–80
T-cell lymphoma	18	7	53.6	20–78
AITL	2	1	63.3	52–78
ALCL	3	1	42.8	25–61
PTCL	13	5	54.3	20–75
NK/T-cell lymphoma	24	16	49.8	28–85
Healthy	22	18	41.9	19–64


*DLBCL, diffuse large B cell lymphoma; FL, Follicular lymphoma; MALToma, mucosa-associated lymphoid tissue lymphoma; MCL, Mantle cell lymphoma; AITL, Angioimmunoblastic T cell lymphoma; ALCL, Anaplastic large cell lymphoma; PTCL, Peripheral T cell lymphoma. NSHL, Nodular sclerosing HL; MCCHL, Mixed Cellularity Classical Hodgkin Lymphoma; NLPHL, nodular lymphocyte predominant Hodgkin’s lymphoma.*

### Identification of a Novel Torque Teno Mini Virus by Viral Metagenomics

Nineteen serum samples of the patients with HL were divided into 4 groups (each group includes 4–5 samples); 252 serum samples of the patients with Non-HL were divided into 51 groups (each group includes 2–5 samples), respectively; 40 serum samples of the patients with non-HL were divided into 8 groups (each group includes 5 samples). For each group above, 50–150 μl equal amount serum was taken from each sample and pooled into one tube. The serum pools were then filtered through a 0.45-μm filter to remove cell and other non-viral particles. To digest unprotected nucleic acids (not in viral capsids), a mixture of DNases (Turbo DNase from Ambion, Waltham, MA, United States, Baseline-ZERO from Epicentre, Chicago, IL, United States, and benzonase from Novagen, Darmstadt, Germany) and RNase (Thermo Fisher Scientific, Waltham, MA, United States) was added to the filtrates, which were enriched in viral particles, and the samples were incubated at 37°C for 60 min (Allander et al., 2001). Viral nucleic acids were then extracted using a QIAamp viral RNA extraction kit (it can purify both viral DNA and RNA from samples) and protected from degradation by the addition of an RNase inhibitor (Thermo Fisher Scientific, Waltham, MA, United States), and stored at -80°C for future processing. Purified viral nucleic acids contain both DNA and RNA. Viral RNA was firstly reverse transcribed into cDNA with SuperScript III reverse transcriptase (Invitrogen) and random primers. Viral DNA and cDNA were amplified and the DNA libraries with index sequences were constructed using Nextera XT Sample Preparation Kit and Nextera XT Index Kit (Illumina, San Diego, CA, United States).

Paired-end reads of 250 bp generated by MiSeq are debarcoded using Illumina vendor software. Using an in-house analysis pipeline running on a 36-nodes Linux cluster, human and bacterial reads were subtracted by mapping to human reference genome hg38 and bacterial nucleotide sequences from nt using bowtie2. Reads are considered duplicates if base positions 5–55 are identical. One random copy of duplicates is kept. Low sequencing quality tails are trimmed using Phred quality score 20 as the threshold. Adaptor and primer sequences are trimmed using the default parameters of VecScreen. The cleaned reads are then de-novo assembled using EnsembleAssembler. The assembled contigs, along with singlets, are aligned to an in-house viral proteome database using BLASTx using *E*-value cutoff of 0.01. The significant hits to viruses are then aligned to an in-house non-virus-non-redundant (NVNR) universal proteome database using DIAMOND. Hits with more significant adjusted *E*-value to NVNR than to viruses are removed. A web-based graphical user interface is used to show the viral matches, along with taxonomy information and processing meta-information. The genome coverage of the target viruses are analyzed by Geneious (Biomatters).

### Amplifications of the Full-Length Torque Teno Mini Virus

Full-length genomes of TTMV were amplified by inverted nested PCR based on two Contigs obtained from the MiSeq analysis and assemble. Based on a 422 bp sequence, two sets of inverse primer pairs, B-OF/B-OR for the 1st round PCR, and B-IF/B-IR (Supplementary Table 1) for the 2nd round PCR, were designed for amplification of the complete genome sequence of one TTMV strains (named TTMV-SH-B) and Sanger sequencing. Based on a 803 bp sequence and a 705 pb sequence, two sets of inverse primer pairs, AC-OF/AC-OR for the 1st round PCR, and AC-IF/AC-IR (Supplementary Table 1) for the 2nd round PCR, were designed for amplification of the complete genome sequence of other TTMV strains (named TTMV-SH-A, TTMV-SH-C1 to TTMV-SH-C9) and Sanger sequencing. The amplification parameters were as follows: 98°C for 2 min, followed by 36 cycles of denaturation at 98°C for 10 s, annealing at 50°C for 1 min, and extension at 72°C for 4 min, and a final extension step of 7 min at 72°C. The second-round cycling parameters were the same as above.

### Phylogenetic Analysis and Sequence Similarity Analysis

To determine the relationship of the novel TTMV to other anelloviruses, phylogenetic analysis was performed based on the ORF1 nucleotide sequences of all 11 new anellovirus isolates and all TTMV strains with full-length genomes in GenBank. And two TTMDV and TTV were used as outgroup. CLUSTALW1.8 was used to perform Sequence alignment with the default settings. A phylogenetic tree with 1000 bootstrap resamples of the alignment data sets was generated using the neighbor-joining method based on the P-distance, pairwise deletion, matrix-based model in MEGA6.0.

### Prevalence Investigation

In order to investigate whether the TTMV is associated with the disease of HL and NHL and present in healthy individuals, DNA from 311 Serum samples (19 patients with HL, 252 patients with NHL and 40 healthy individuals) was extracted using a TAKARA MiniBEST Viral RNA/DNA Extraction kit Ver 5.0 according to the manufacturer’s instructions. Nested primers were designed and based on common sequence of all the 11 new strains. Primer pair Out-F/Out-R was used for the 1st round PCR and the product is 1621 bp. Primer pair In-F/In-R was used for the 2nd round PCR and the product is 1191 bp. The first round amplification conditions were as follows: 98°C for 2 min, followed by 40 cycles of denaturation at 98°C for 10 s, annealing at 55°C for 30 s, and extension at 72°C for 2 min, and a final extension step of 7 min at 72°C. The second-round cycling conditions were the same as first round. PCR products were resolved by electrophoresis on 1% agarose gels. Fisher’s exact test was used to analyze the data by SPSS 17.0 (IBM, New York, NY, United States) and the significance level was set at 0.05.

## Results

### Discovery of a Novel TTMV

**FIGURE 1:**
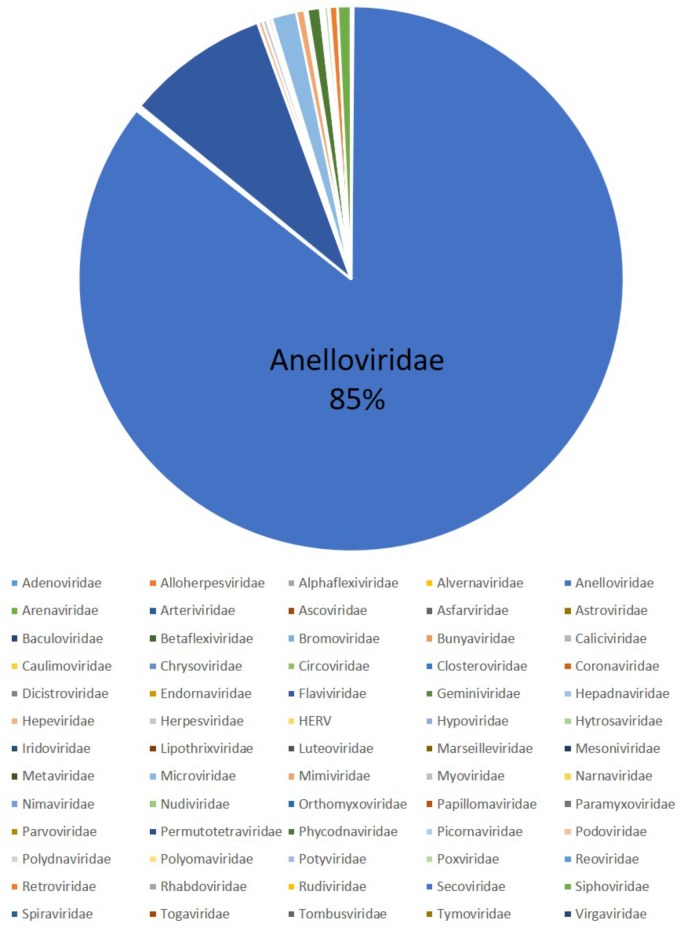
Viruses detected through viral metagenomics. 65 virus families are detected in our samples as follows: Adenoviridae (141), Alloherpesviridae (17), Alphaflexiviridae (3), Alvernaviridae (1), Anelloviridae (101130), Arenaviridae (3), Arteriviridae (18), Ascoviridae (68), Asfarviridae (5), Astroviridae (5), Baculoviridae (133), Betaflexiviridae (10), Bromoviridae (3), Bunyaviridae (17), Caliciviridae (22), Caulimoviridae (61), Chrysoviridae (1), Circoviridae (19), Closteroviridae (13), Coronaviridae (32), Dicistroviridae (1), Endornaviridae (2), Flaviviridae (10038), Geminiviridae (5), Hepadnaviridae (45), Hepeviridae (305), Herpesviridae (316), Human endogenous retroviruses (HERV, 123), Hypoviridae (2), Hytrosaviridae (4), Iridoviridae (187), Lipothrixviridae (4), Luteoviridae (2), Marseilleviridae (42), Mesoniviridae (3), Metaviridae (1), Microviridae (1741), Mimiviridae (581), Myoviridae (15), Narnaviridae (11), Nimaviridae (7), Nudiviridae (9), Orthomyxoviridae (3), Papillomaviridae (65), Paramyxoviridae (20), Parvoviridae (60), Permutotetraviridae (15), Phycodnaviridae (879), Picornaviridae (42), Podoviridae (160), Polydnaviridae (42), Polyomaviridae (27), Potyviridae (26), Poxviridae (272), Reoviridae (98), Retroviridae (544), Rhabdoviridae (11), Rudiviridae (4), Secoviridae (5), Siphoviridae (916), Spiraviridae (7958), Togaviridae (3), Tombusviridae (1), Tymoviridae (1), and Virgaviridae (3).

Nineteen HL, 252 non- HL, and 40 healthy donor serum samples were analyzed using a viral metagenomics approach (**Table 1**). And 118,348 viral sequence reads were obtained, which consist of 65 virus families (**Figure 1** and Supplementary Table 2). Among them top 10 sequences cluster into Anelloviridae (85.45%), Flaviviridae (8.48%), Microviridae (1.47%), Siphoviridae (0.77%), Phycodnaviridae (0.74%), Mimiviridae (0.49%), Retroviridae (0.46%), Herpesviridae (0.27%), Hepeviridae (0.26%), and Poxviridae (0.23%). We found a novel species of TTMV virus in 3 out of 19 human HL serum samples, but not in 252 non-Hodgkin lymphoma and 40 healthy donor serum samples (**Table 2**).

**Table 2:** Relationship between Hodgkin’s lymphoma and TTMV-SH.

Group	*n*^∗^	Total	% with TTMV-SH	*P^∗∗^*
**Lymphoma**				
Positive	3	271	1.1	>0.5
Negative	0	40	0.0	
**Hodgkin’s lymphoma**				
Positive	3	19	15.8	<0.01
Negative	0	292	0	
Hodgkin’s lymphoma positive	3	19	15.8	<0.05
Healthy	0	40	0	


*^∗^ “n” means TTMV-SH positive sample number. ^∗∗^*P*-value was made using Fisher’s exact test.*

**FIGURE 2:**
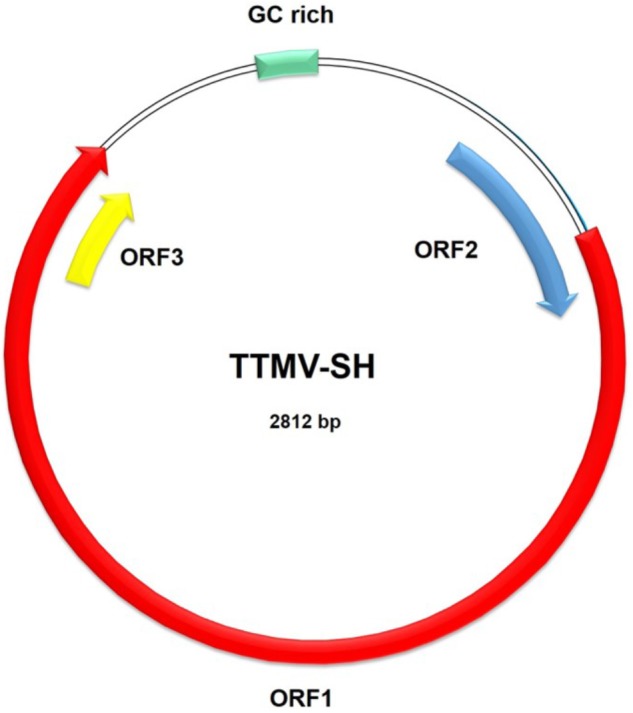
The genome organization of novel TTMV virus TTMV-SH. The genome length of TTMV-SH is 2812nt. The genome organization contains three open reading frames (ORFs) and one GC rich region. ORF1 is 1962nt in length and encodes 653 amino acids. ORF2 and ORF3 encodes 123 amino acids and 63 amino acids, respectively.

The complete genome of 11 isolates of the torque teno mini viruses were obtained from three HL, which all belongs to pathologic subtype nodular sclerosing Hodgkin’s lymphoma (NSHL, Supplementary Table 3), serum samples by viral sequences assembly and PCR. One TTMV isolate from sample 5 and named TTMV-SH-A (GenBank accession No. KY462762); one TTMV isolate was obtained from sample 6 and named TTMV-SH-B (GenBank accession No. KY462769), and the other 9 isolates were obtained from sample 16 and named TTMV-SH-C1 to TTMV-SH-C9 (GenBank accession No. KY462758-61, KY462763-66 and KY462768). The genome length of all the 11 isolates is 2812nt (**Figure 2** and Supplementary Table 4). All the 11 anellovirus isolates share similar genome organization, which contains three open reading frames (ORFs) and one GC rich region (**Figure 2**). ORF1 of all the 11 isolates is 1962nt in length and encodes 653 amino acids (Supplementary Table 4). ORF2 and ORF3 of all the 11 isolates encodes 123 amino acids and 63 amino acids, respectively (Supplementary Table 4). The ORF2 of the 11 isolates has a 31-amino acid (aa) extension at N-terminus compared to most reference strains (Supplementary Figure 1), while their N-terminal ORF3 is 50 amino acids shorter than most reference strains (Supplementary Figure 2).

**FIGURE 3:**
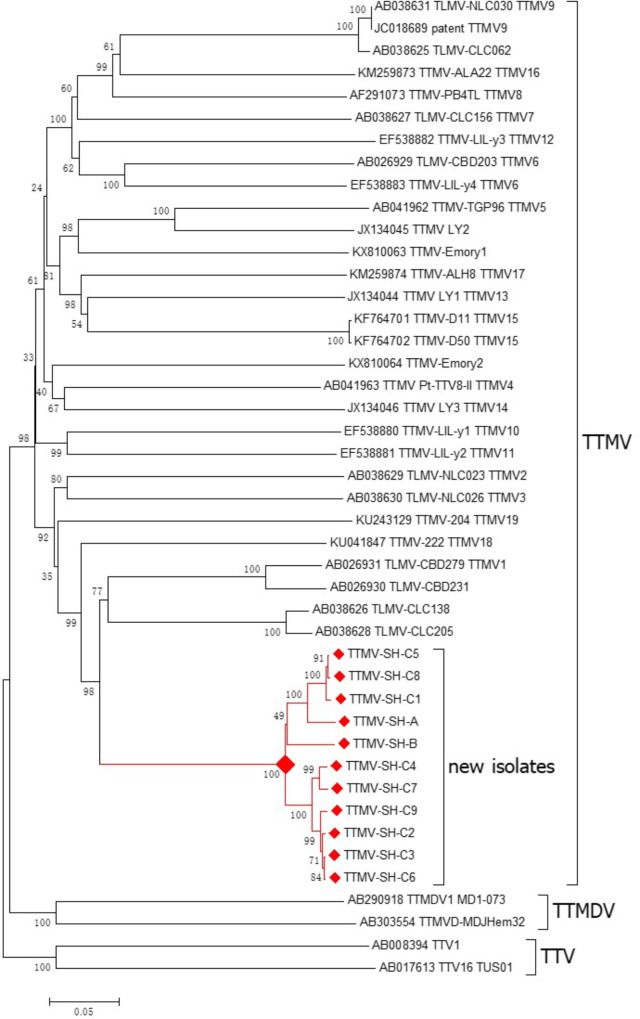
Phylogenetic tree based on the nucleotide sequence of ORF1 was constructed using the neighbor-joining method with nucleotide p distances and 1,000 bootstrap replicates in the Molecular Evolutionary Genetics Analysis program (MEGA, version 6.0, United States). All TTMV strains with full genome uploaded to GenBank were used as references, Two TTMDV strains and two TTMV strains were used as outgroup. Bootstrap values are indicated at each branching point. The red solid diamond indicates the novel TTMV specie for 11 isolates from three patients.

Phylogenetic analysis indicated that the 11 isolates cluster into one group (**Figure 3**), sharing over 94.3% genome sequences similarity (Supplementary Table 5) and 94.2–99.9% ORF1 nucleotide sequence similarity (Supplementary Figure 3). The ORF1 nucleotide sequences divergence among the 11 isolates is 0.1–7.0% (Supplementary Figure 3), which indicates they belong to one species.

Isolates of TTMV-SHC1 to TTMV-SHC9 were obtained from one sample. Among them, three isolates, TTMV-SH-C1, TTMV-SH-C5, and TTMV-SH-C8, cluster into one group and sharing 99.5–99.8% ORF1 nucleotide sequence similarity; the other 6 isolates cluster into the other group and sharing 98.7–99.9% ORF1 nucleotide sequence similarity; and the two group sharing only 94.2–95.1% ORF1 nucleotide sequence similarity.

Phylogenetic analysis also indicated that the 11 isolates cluster with 4 torque teno mini viruses discovered from human (TLMV-CBD279, TLMV-CBD231, TLMV-CLC138 and TLMV-CLC205, **Figure 3**). The 11 isolates share 69.4–70.4% genome sequences similarity and 60.1–62% ORF1 nucleotide sequences similarity with the four reference isolates (Supplementary Table 6). The ORF1 nucleotide sequences divergence between the 13 isolates and the 4 reference isolates is 39.2–44.1% (Supplementary Table 6), which indicates that the 11 new isolates belong to a novel species.

TTMV strain TTMV-SH-C4 share the highest ORF1 nucleotide sequence similarity (62%) reference strain TLMV-CBD231 among all the 13 strains and 4 reference strains (Supplementary Table 6). Strain TTMV-SH-C4 share 53.5–70.4% genome sequence similarity, 46.5–62% ORF1 nucleotide sequence similarity, 32.2–63.1% ORF2 nucleotide sequence similarity, 30.4–77.7% ORF3 nucleotide sequence similarity, 37.3–60% ORF1 amino acid sequence similarity, 24.5–58.3% ORF2 amino acid sequence similarity and 25.9–73.2% ORF3 amino acid sequence similarity with all reference TTMV strains, respectively (Supplementary Table 7).

### Prevalence

Prevalence investigation showed that all of the Non-Hodgkin lymphoma and all the 40 healthy samples were negative for TTMV-SH. TTMV-SH prevalence is 15.8% (3/19) in HL (**Table 2**) and 30% (3/10) in nodular sclerosing HL (Supplementary Table 8), but not in other pathologic subtypes of HL. All the HL and the 40 healthy samples are negative for lymphoma associated viruses like HIV, HTLV and EB virus. These three viruses prevalence are 1.7% (5/292), 0.7% (2/292), and 0.3% (1/292) in non-Hodgkin lymphoma (Supplementary Table 8).

The difference in the TTMV-SH prevalence between HL and healthy samples was statistically significant (*p* < 0.05, **Table 2**), and there is no statistical difference between HIV, HTLV and EB virus prevalence and healthy samples (Supplementary Table 8), suggesting that detection of TTMV-SH may be associated with HL, although this finding requires further investigation.

## Discussion

Hodgkin’s lymphomas and the NHL are two types of lymphomas. The mainly risk factors for lymphomas are infectious virus, especially Epstein–Barr virus, HIV, hepatitis C virus, and human T-lymphotropic virus (Stewart and Wild, 2014; Gloghini et al., 2017; Linke-Serinsoz et al., 2017; Pozzato et al., 2017). Anelloviruses are associated with hepatitis, respiratory diseases, cancer, hematological, periodontitis, and autoimmune disorders (Galmes et al., 2013; Spandole et al., 2015; Zhang et al., 2016, 2017). Other groups have also reported the discovery of anellovirus sequences in a human melanoma and in lymph nodes of patients with Hodgkin lymphoma. Two new human anellovirus species (TTMV Emory1 and TTMV Emory2) were identified in one brain tumor metastasized from a skin melanoma (Ng et al., 2017). In another report, although the frequencies of TTV DNA were not significantly different between EBV negative (54%) and EBV positive (50%) nodes, it was observed that the group of young adults (15–34 years, *n* = 19) showed the lowest EBV frequency (21%) but the highest TTV occurrence (60%). This may suggest an involvement of TTV infection in the pathogenesis of HL in young adults (Figueiredo et al., 2007). However, due to the limited sample size, it is unclear if the anelloviruses contributed to tumor formation (Figueiredo et al., 2007; Ng et al., 2017).

In our study, viral metagenomics were performed in lymphomas, and 65 virus families were detected. Among them, three virus families, Microviridae, Siphoviridae, and Mimiviridae, which infect bacteria, were detected in human serum. This phenomenon might be caused by concurrent infection of bacteria, though contamination introduced during skin sampling and other processes could not be ruled out. The detection of plant-infecting virus family Phycodnaviridae was mostly likely due to contamination.

We discovered a new human Torque Teno Mini Virus TTMV-SH from the serum of three HL patients. Phylogenetic analysis indicated that this newly characterized genome belonged to genus Betatorquevirus. In addition, we investigated the prevalence of TTMV-SH in HL, NHL patients and healthy people. The healthy and NHL samples were all negative, but three of nineteen HL (3/10 NSHL) patient samples were positive. And one patient was apparently infected by a mixture of two groups of TTMV-SH with 9 isolates. Mixed infections may be caused by concurrent or sequential exposure to different subtypes or genotypes, for example, due to multiple blood transfusions or reuse of inadequately sterilized medical equipment. Unfortunately, the epidemiological data on the patient with mixed infections were incomplete and could not be used to draw conclusions regarding causes of the mixed infections.

The TTMV-SH prevalence in HL group was significantly higher than in the healthy group (*p* < 0.05, **Table 2**) suggesting that TTMV-SH may be more likely to be found in HL patients, especially in NSHL than healthy individuals and NHL patients. HIV, HTLV and EBV, the three lymphoma-associated viruses, were not detected in both HL and healthy samples in this study. The lack of EBV is surprising since EBV is known as the causative agent for a substantial cases of HL (Stewart and Wild, 2014). We checked the sequence data of each of the 19 HL serum samples, but found no EB virus DNA. The lack of EBV may be caused by limited sample number or unknown reasons. However, due to limited quantity of the HL patients’ serum, the association between HL (especially NSHL) and TTMV-SH needs further study.

## Conclusion

The complete genomes of Torque Teno Mini Virus (TTMV-SH) in the serum of the lymphoma patients were sequenced and characterized. Phylogenetic analysis suggests that TTMV-SH belonged to a novel anellovirus species divergent from those anellovirus available in GenBank. The molecular epidemiology shows the TTMV may be related to HLs, especially NSHL.

## Author Contributions

XF, YX, and SP designed and wrote the manuscript. SP and TY performed the experiment and analyzed the data. HW, RL, and YW collected the serum samples.

## Conflict of Interest Statement

The authors declare that the research was conducted in the absence of any commercial or financial relationships that could be construed as a potential conflict of interest.
