# Supplementary material for: Identification of a Torque Teno Mini Virus (TTMV) in Hodgkin’s Lymphoma Patients
Source: Front Microbiol. 2018 Jul 26;9:1680. doi: 10.3389/fmicb.2018.01680 (PMC6070622; doi:10.3389/fmicb.2018.01680)
Supplement: Supplementary file 3 [file Image_3.PDF]

Percent Similarity

Divergence

	1	2	3	4	5	6	7	8	9	10	11		
1		93.4	95.3	94.2	93.9	93.8	95.7	94.1	94.1	95.6	94.3	1	SH-A
2	6.9		93.5	94.2	93.6	93.8	93.3	93.7	94.0	93.4	94.2	2	SH-B
3	4.9	6.8		94.4	94.2	94.2	99.5	94.3	94.8	99.7	94.5	3	SH-C1
4	6.0	6.1	5.8		99.2	99.1	94.8	99.1	98.7	94.7	99.9	4	SH-C2
5	6.3	6.7	6.1	0.8		99.4	94.6	99.8	98.8	94.5	99.1	5	SH-C3
6	6.5	6.5	6.1	0.9	0.6		94.5	99.3	98.7	94.5	99.0	6	SH-C4
7	4.5	7.0	0.5	5.5	5.6	5.7		94.8	95.1	99.8	94.9	7	SH-C5
8	6.2	6.6	5.9	0.9	0.2	0.7	5.5		98.9	94.6	99.2	8	SH-C6
9	3.1	6.3	5.5	1.3	1.2	1.3	5.1	1.1		95.1	98.8	9	SH-C7
10	4.6	7.0	0.3	5.5	5.7	5.7	0.2	5.6	5.1		94.8	10	SH-C8
11	5.9	6.1	5.7	0.1	0.9	1.0	5.3	0.8	1.2	5.4		11	SH-C9
	1	2	3	4	5	6	7	8	9	10	11		

Supplementary Figure 3. ORF1 nucleotide sequences similarity and divergence between the 11 new isolates.
