# Supplementary material for: Identification of a Torque Teno Mini Virus (TTMV) in Hodgkin’s Lymphoma Patients
Source: Front Microbiol. 2018 Jul 26;9:1680. doi: 10.3389/fmicb.2018.01680 (PMC6070622; doi:10.3389/fmicb.2018.01680)
Supplement: Supplementary file 4 [file Table_1.DOCX]

**Supplementary table 1. Primers for amplification genome**

| Primers | Primer sequence (5’-3’) | Relative position |
| --- | --- | --- |
| B-OF | G*A*ATGGCTGAGTTTACCCCGCCAGA | 184-208 |
| B-OR | G*G*AAGTAAGTGTAAAAATAGGGAAGTAAGG | 65-94 |
| B-IF | ACACTGACTCGGCTGCTTTAGGGCA | 225-248 |
| B-IR | ACCGTTTGTGGTCGGCAATTCTAAT | 30-54 |
| AC-OF | G*G*AGACCTAGACAAACTATTCGAAG | 580-604 |
| AC-OR | G*G*AGGCATGGTAGATTGTTTTTCTT | 487-511 |
| AC-IF | TATGATGAATATACAGCTTTTAGAA | 861-885 |
| AC-IR | GCTGTAAAATAAAATATTATTTTAG | 326-350 |
| Out-F | A*C*AGCAAAAATGGCTTAGAAAACCA | 280-404 |
| Out-R | T*A*GTGCTATGTTTTGTGTGGTGTAT | 19761998 |
| In-F | GGAGAAGACCACGGAGATGGATTCG | 550-574 |
| In-R | GTATTTGTTCATTTTGCGGTAAGGA | 1716-1740 |

Note: “*” means phosphoroth-ioation.
