# Supplementary material for: Identification of a Torque Teno Mini Virus (TTMV) in Hodgkin’s Lymphoma Patients
Source: Front Microbiol. 2018 Jul 26;9:1680. doi: 10.3389/fmicb.2018.01680 (PMC6070622; doi:10.3389/fmicb.2018.01680)
Supplement: Supplementary file 5 [file Table_2.DOCX]

**Supplementary table 2. Viruses detected through viral metagenomics in different pools**

| **Viruses** | **Reads** | | | | **Percentage (%)** | | | |
| --- | --- | --- | --- | --- | --- | --- | --- | --- |
|  | **NHL** | **HL** | **Healthy** | **Sum** | **NHL** | **HL** | **Healthy** | **Sum** |
| Adenoviridae | 102 | 36 | 3 | 141 | 0.09 | 0.64 | 0.13 | 0.12 |
| Alloherpesviridae | 16 | 0 | 1 | 17 | 0.01 | 0.00 | 0.04 | 0.01 |
| Alphaflexiviridae | 3 | 0 | 0 | 3 | 0.00 | 0.00 | 0.00 | 0.00 |
| Alvernaviridae | 1 | 0 | 0 | 1 | 0.00 | 0.00 | 0.00 | 0.00 |
| Anelloviridae | 96028 | 5045 | 57 | 101130 | 84.31 | 90.04 | 2.43 | 83.00 |
| Arenaviridae | 3 | 0 | 0 | 3 | 0.00 | 0.00 | 0.00 | 0.00 |
| Arteriviridae | 15 | 0 | 3 | 18 | 0.01 | 0.00 | 0.13 | 0.01 |
| Ascoviridae | 63 | 2 | 3 | 68 | 0.06 | 0.04 | 0.13 | 0.06 |
| Asfarviridae | 5 | 0 | 0 | 5 | 0.00 | 0.00 | 0.00 | 0.00 |
| Astroviridae | 5 | 0 | 0 | 5 | 0.00 | 0.00 | 0.00 | 0.00 |
| Baculoviridae | 112 | 18 | 3 | 133 | 0.10 | 0.32 | 0.13 | 0.11 |
| Betaflexiviridae | 9 | 1 | 0 | 10 | 0.01 | 0.02 | 0.00 | 0.01 |
| Bromoviridae | 3 | 0 | 0 | 3 | 0.00 | 0.00 | 0.00 | 0.00 |
| Bunyaviridae | 17 | 0 | 0 | 17 | 0.01 | 0.00 | 0.00 | 0.01 |
| Caliciviridae | 17 | 5 | 0 | 22 | 0.01 | 0.09 | 0.00 | 0.02 |
| Caulimoviridae | 52 | 6 | 3 | 61 | 0.05 | 0.11 | 0.13 | 0.05 |
| Chrysoviridae | 0 | 0 | 1 | 1 | 0.00 | 0.00 | 0.04 | 0.00 |
| Circoviridae | 12 | 5 | 2 | 19 | 0.01 | 0.09 | 0.09 | 0.02 |
| Closteroviridae | 13 | 0 | 0 | 13 | 0.01 | 0.00 | 0.00 | 0.01 |
| Coronaviridae | 27 | 5 | 0 | 32 | 0.02 | 0.09 | 0.00 | 0.03 |
| Dicistroviridae | 1 | 0 | 0 | 1 | 0.00 | 0.00 | 0.00 | 0.00 |
| Endornaviridae | 2 | 0 | 0 | 2 | 0.00 | 0.00 | 0.00 | 0.00 |
| Flaviviridae | 10026 | 5 | 7 | 10038 | 8.80 | 0.09 | 0.30 | 8.24 |
| Geminiviridae | 4 | 0 | 1 | 5 | 0.00 | 0.00 | 0.04 | 0.00 |
| Hepadnaviridae | 39 | 2 | 4 | 45 | 0.03 | 0.04 | 0.17 | 0.04 |
| Hepeviridae | 303 | 0 | 2 | 305 | 0.27 | 0.00 | 0.09 | 0.25 |
| Herpesviridae | 280 | 20 | 16 | 316 | 0.25 | 0.36 | 0.68 | 0.26 |
| HERV | 108 | 13 | 2 | 123 | 0.09 | 0.23 | 0.09 | 0.10 |
| Hypoviridae | 2 | 0 | 0 | 2 | 0.00 | 0.00 | 0.00 | 0.00 |
| Hytrosaviridae | 4 | 0 | 0 | 4 | 0.00 | 0.00 | 0.00 | 0.00 |
| Iridoviridae | 179 | 0 | 8 | 187 | 0.16 | 0.00 | 0.34 | 0.15 |
| Lipothrixviridae | 2 | 0 | 2 | 4 | 0.00 | 0.00 | 0.09 | 0.00 |
| Luteoviridae | 2 | 0 | 0 | 2 | 0.00 | 0.00 | 0.00 | 0.00 |
| Marseilleviridae | 29 | 11 | 2 | 42 | 0.03 | 0.20 | 0.09 | 0.03 |
| Mesoniviridae | 3 | 0 | 0 | 3 | 0.00 | 0.00 | 0.00 | 0.00 |
| Metaviridae | 1 | 0 | 0 | 1 | 0.00 | 0.00 | 0.00 | 0.00 |
| Microviridae | 27 | 4 | 1710 | 1741 | 0.02 | 0.07 | 72.77 | 1.43 |
| Mimiviridae | 528 | 10 | 43 | 581 | 0.46 | 0.18 | 1.83 | 0.48 |
| Myoviridae | 13 | 0 | 2 | 15 | 0.01 | 0.00 | 0.09 | 0.01 |
| Narnaviridae | 9 | 0 | 2 | 11 | 0.01 | 0.00 | 0.09 | 0.01 |
| Nimaviridae | 7 | 0 | 0 | 7 | 0.01 | 0.00 | 0.00 | 0.01 |
| Nudiviridae | 7 | 2 | 0 | 9 | 0.01 | 0.04 | 0.00 | 0.01 |
| Orthomyxoviridae | 1 | 2 | 0 | 3 | 0.00 | 0.04 | 0.00 | 0.00 |
| Papillomaviridae | 65 | 0 | 0 | 65 | 0.06 | 0.00 | 0.00 | 0.05 |
| Paramyxoviridae | 16 | 0 | 4 | 20 | 0.01 | 0.00 | 0.17 | 0.02 |
| Parvoviridae | 56 | 4 | 0 | 60 | 0.05 | 0.07 | 0.00 | 0.05 |
| Permutotetraviridae | 13 | 0 | 2 | 15 | 0.01 | 0.00 | 0.09 | 0.01 |
| Phycodnaviridae | 751 | 51 | 77 | 879 | 0.66 | 0.91 | 3.28 | 0.72 |
| Picornaviridae | 40 | 1 | 1 | 42 | 0.04 | 0.02 | 0.04 | 0.03 |
| Podoviridae | 138 | 16 | 6 | 160 | 0.12 | 0.29 | 0.26 | 0.13 |
| Polydnaviridae | 37 | 3 | 2 | 42 | 0.03 | 0.05 | 0.09 | 0.03 |
| Polyomaviridae | 26 | 0 | 1 | 27 | 0.02 | 0.00 | 0.04 | 0.02 |
| Potyviridae | 24 | 0 | 2 | 26 | 0.02 | 0.00 | 0.09 | 0.02 |
| Poxviridae | 238 | 22 | 12 | 272 | 0.21 | 0.39 | 0.51 | 0.22 |
| Reoviridae | 74 | 21 | 3 | 98 | 0.06 | 0.37 | 0.13 | 0.08 |
| Retroviridae | 465 | 43 | 36 | 544 | 0.41 | 0.77 | 1.53 | 0.45 |
| Rhabdoviridae | 10 | 1 | 0 | 11 | 0.01 | 0.02 | 0.00 | 0.01 |
| Rudiviridae | 3 | 0 | 1 | 4 | 0.00 | 0.00 | 0.04 | 0.00 |
| Secoviridae | 5 | 0 | 0 | 5 | 0.00 | 0.00 | 0.00 | 0.00 |
| Siphoviridae | 756 | 37 | 123 | 916 | 0.66 | 0.66 | 5.23 | 0.75 |
| Spiraviridae | 5 | 0 | 0 | 5 | 0.00 | 0.00 | 0.00 | 0.00 |
| Togaviridae | 3 | 0 | 0 | 3 | 0.00 | 0.00 | 0.00 | 0.00 |
| Tombusviridae | 1 | 0 | 0 | 1 | 0.00 | 0.00 | 0.00 | 0.00 |
| Tymoviridae | 1 | 0 | 0 | 1 | 0.00 | 0.00 | 0.00 | 0.00 |
| Virgaviridae | 3 | 0 | 0 | 3 | 0.00 | 0.00 | 0.00 | 0.00 |
| Sum | 110810 | 5391 | 2147 | 121848 | 100 | 100 | 100 | 100 |

Note: HL means Hodgkin’s lymphoma, NHL means non-Hodgkin lymphomas, and Healthy means healthy donor, HERV means human endogenous retroviruses.
