# Supplementary material for: Identification of a Torque Teno Mini Virus (TTMV) in Hodgkin’s Lymphoma Patients
Source: Front Microbiol. 2018 Jul 26;9:1680. doi: 10.3389/fmicb.2018.01680 (PMC6070622; doi:10.3389/fmicb.2018.01680)
Supplement: Supplementary file 6 [file Table_3.DOCX]

**Supplementary Table 3. Hodgkin's lymphoma pathologic subtypes and TTMV DNA detection in patients' serum**

| **No.** | **Gender** | **Age** | **Pathologic subtypes** | **Detection of TTMV DNA** |
| --- | --- | --- | --- | --- |
| 1 | male | 25 | NSHL | - |
| 2 | male | 42 | MCCHL | - |
| 3 | female | 17 | MCCHL | - |
| 4 | female | 26 | NSHL | - |
| 5 | female | 17 | NSHL | **+** |
| 6 | male | 39 | NSHL | **+** |
| 7 | female | 20 | NSHL | - |
| 8 | female | 29 | NLPHL | - |
| 9 | male | 31 | MCCHL | - |
| 10 | female | 54 | MCCHL | - |
| 11 | female | 32 | NSHL | - |
| 12 | male | 37 | MCCHL | - |
| 13 | male | 37 | MCCHL | - |
| 14 | female | 28 | NSHL | - |
| 15 | female | 41 | NSHL | - |
| 16 | male | 15 | NSHL | + |
| 17 | male | 15 | MCCHL | - |
| 18 | male | 63 | MCCHL | - |
| 19 | female | 36 | NSHL | - |

Note: NSHL: nodular sclerosing Hodgkin Lymphoma; MCCHL: mixed cellularity classical Hodgkin Lymphoma; NLPHL: nodular lymphocyte predominant Hodgkin's lymphoma
