# Supplementary material for: Identification of a Torque Teno Mini Virus (TTMV) in Hodgkin’s Lymphoma Patients
Source: Front Microbiol. 2018 Jul 26;9:1680. doi: 10.3389/fmicb.2018.01680 (PMC6070622; doi:10.3389/fmicb.2018.01680)
Supplement: Supplementary file 7 [file Table_4.DOCX]

**Supplementary table 4. Genome and ORFs length of the 11 TTMV-SH isolates and reference strains**

| **TTMV strain (GenBank**  **accession NO.)** | **Nucleotides** | | | | **Amino acids** | | | **Percentage of ORF1 in genome** |
| --- | --- | --- | --- | --- | --- | --- | --- | --- |
|  | **Genome** | **ORF1** | **ORF2** | **ORF3** | **ORF1** | **ORF2** | **ORF3** |  |
| TTMV-SH-A | 2812 | 1962 | 372 | 192 | 653 | 123 | 63 | 69.8 |
| TTMV-SH-B | 2812 | 1962 | 372 | 192 | 653 | 123 | 63 | 69.8 |
| TTMV-SH-C1 | 2812 | 1962 | 372 | 192 | 653 | 123 | 63 | 69.8 |
| TTMV-SH-C2 | 2812 | 1962 | 372 | 192 | 653 | 123 | 63 | 69.8 |
| TTMV-SH-C3 | 2812 | 1962 | 372 | 192 | 653 | 123 | 63 | 69.8 |
| TTMV-SH-C4 | 2812 | 1962 | 372 | 192 | 653 | 123 | 63 | 69.8 |
| TTMV-SH-C5 | 2812 | 1962 | 372 | 192 | 653 | 123 | 63 | 69.8 |
| TTMV-SH-C6 | 2812 | 1962 | 372 | 192 | 653 | 123 | 63 | 69.8 |
| TTMV-SH-C7 | 2812 | 1962 | 372 | 192 | 653 | 123 | 63 | 69.8 |
| TTMV-SH-C8 | 2812 | 1962 | 372 | 192 | 653 | 123 | 63 | 69.8 |
| TTMV-SH-C9 | 2812 | 1962 | 372 | 192 | 653 | 123 | 63 | 69.8 |
| TLMV-CBD279 (AB026931) | 2856 | 1989 | 276 | 393 | 662 | 91 | 130 | 69.6 |
| TLMV-CBD231 (AB026930) | 2860 | 1992 | 276 | 393 | 663 | 91 | 130 | 69.7 |
| TLMV-CLC138 (AB038626) | 2841 | 1992 | 279 | 396 | 663 | 92 | 131 | 70.1 |
| TLMV-CLC205 (AB038628) | 2841 | 1992 | 279 | 396 | 663 | 92 | 131 | 70.1 |
| TLMV-NLC023 (AB038629) | 2765 | 1887 | 270 | 387 | 628 | 89 | 128 | 68.2 |
| TLMV-NLC026 (AB038630) | 2897 | 1986 | 330 | 396 | 661 | 109 | 131 | 68.6 |
| TTMV-Pt-TTV8-II (AB041963) | 2785 | 1965 | 276 | -* | 654 | 91 | -* | 70.6 |
| TTMV-TGP96 (AB041962) | 2908 | 2016 | 300 | 339 | 671 | 99 | 112 | 69.3 |
| TTMV-LY2 (JX134045) | 2979 | 2001 | 300 | 339 | 666 | 99 | 112 | 67.2 |
| TLMV-CBD203 (AB026929) | 2897 | 1971 | 294 | 423 | 656 | 97 | 140 | 68.0 |
| TTMV-LIL-y4 (EF538883) | 2797 | 1947 | 291 | -* | 648 | 96 | -* | 69.6 |
| TLMV-CLC156 (AB038627) | 2952 | 1998 | 303 | -* | 665 | 100 | -* | 67.7 |
| TTMV-PB4TL (AF291073) | 2910 | 2028 | 300 | -* | 675 | 99 | -* | 69.7 |
| TLMV-NLC030 (AB038631) | 2915 | 2052 | 303 | 348 | 683 | 100 | 115 | 70.4 |
| TLMV-CLC062 (AB038625) | 2915 | 2052 | 318 | 348 | 683 | 105 | 115 | 70.4 |
| Patent EP2653562 (JC018689) | 2915 | 2052 | 402 | 348 | 683 | 133 | 115 | 70.4 |
| TTMV-LIL-y1 (EF538880) | 2887 | 1953 | 324 | 345 | 650 | 107 | 114 | 67.6 |
| TTMV-LIL-y2 (EF538881) | 2871 | 1989 | 312 | 570 | 662 | 103 | 189 | 69.3 |
| TTMV-LIL-y3 (EF538882) | 2912 | 1986 | 300 | 354 | 661 | 99 | 117 | 68.2 |
| TTMV-LY1 (JX134044) | 2912 | 2019 | 291 | 303 | 672 | 96 | 100 | 69.3 |
| TTMV-LY3 (JX134046) | 2912 | 1977 | 291 | 387 | 658 | 96 | 128 | 67.9 |
| TTMV-D11 (KF764701) | 2853 | 1989 | 285 | 381 | 662 | 94 | 126 | 69.7 |
| TTMV-D50 (KF764702) | 2853 | 1989 | 285 | 381 | 662 | 94 | 126 | 69.7 |
| TTMV-ALA22 (KM259873) | 2914 | 2025 | 306 | 405 | 674 | 101 | 134 | 69.5 |
| TTMV-ALH8 (KM259874) | 2855 | 1971 | 270 | -* | 656 | 89 | -* | 69.0 |
| TTMV-222 (KU041847) | 2803 | 1941 | 267 | 273 | 646 | 88 | 90 | 69.2 |
| TTMV-Emory1 (KX810063) | 2830 | 1992 | 303 | 339 | 663 | 100 | 112 | 70.4 |
| TTMV-Emory2 (KX810064) | 2938 | 2031 | 333 | 240 | 676 | 110 | 79 | 69.1 |

*There is no ORF3 in these TTMV isolate Pt-TTV8-II, TTMV-LIL-y4, TLMV-CLC156, TTMV-PB4TL and ALH8
