# Supplementary material for: Identification of a Torque Teno Mini Virus (TTMV) in Hodgkin’s Lymphoma Patients
Source: Front Microbiol. 2018 Jul 26;9:1680. doi: 10.3389/fmicb.2018.01680 (PMC6070622; doi:10.3389/fmicb.2018.01680)
Supplement: Supplementary file 8 [file Table_5.DOCX]

**Supplementary table 5. Genome sequence similarity between the 11 TTMV-SH isolates**

|  |  | **1** | **2** | **3** | **4** | **5** | **6** | **7** | **8** | **9** | **10** | **11** |
| --- | --- | --- | --- | --- | --- | --- | --- | --- | --- | --- | --- | --- |
| SH-A | **1** | *** |  |  |  |  |  |  |  |  |  |  |
| SH-B | **2** | 94.5 | *** |  |  |  |  |  |  |  |  |  |
| SH-C1 | **3** | 95.7 | 94.5 | *** |  |  |  |  |  |  |  |  |
| SH-C2 | **4** | 94.9 | 94.9 | 95 | *** |  |  |  |  |  |  |  |
| SH-C3 | **5** | 94.7 | 94.6 | 94.9 | 99.4 | *** |  |  |  |  |  |  |
| SH-C4 | **6** | 94.6 | 94.7 | 94.9 | 99.4 | 99.6 | *** |  |  |  |  |  |
| SH-C5 | **7** | 96 | 94.3 | 99.7 | 95.2 | 95.2 | 95.1 | *** |  |  |  |  |
| SH-C6 | **8** | 94.8 | 94.6 | 95 | 99.4 | 99.9 | 99.5 | 95.3 | *** |  |  |  |
| SH-C7 | **9** | 94.8 | 94.8 | 95.3 | 99.1 | 99.1 | 99.1 | 95.5 | 99.3 | *** |  |  |
| SH-C8 | **10** | 95.9 | 94.4 | 99.8 | 95.2 | 95.1 | 95.1 | 99.9 | 95.2 | 95.5 | *** |  |
| SH-C9 | **11** | 95 | 94.9 | 95.1 | 99.9 | 99.3 | 99.3 | 95.3 | 99.4 | 99.1 | 95.3 | *** |
|  |  | **1** | **2** | **3** | **4** | **5** | **6** | **7** | **8** | **9** | **10** | **11** |

Note: Bold number means respective virus.

“***” means sequence similarity between the same TTMV isolates.
