# Supplementary material for: Identification of a Torque Teno Mini Virus (TTMV) in Hodgkin’s Lymphoma Patients
Source: Front Microbiol. 2018 Jul 26;9:1680. doi: 10.3389/fmicb.2018.01680 (PMC6070622; doi:10.3389/fmicb.2018.01680)
Supplement: Supplementary file 9 [file Table_6.DOCX]

**Supplementary table 6. Nucleotide sequence similarity and divergence between the 11 TTMV-SH isolates and 4 reference TTMV strains**

| **TTMV-SH isolate** | **similarity** | | | | | | | | **Divergence** | | | |
| --- | --- | --- | --- | --- | --- | --- | --- | --- | --- | --- | --- | --- |
|  | **Genome** | | | | **ORF1** | | | | | | | |
|  | CBD279 | CBD231 | CLC138 | CLC205 | CBD279 | CBD231 | CLC138 | CLC205 | CBD279 | CBD231 | CLC138 | CLC205 |
| SH-A | 70 | 70.1 | 70.1 | 70.1 | 60.7 | 61.1 | 60.5 | 60.1 | 44 | 42.4 | 41.1 | 40.6 |
| SH-B | 69.5 | 69.4 | 70.4 | 70.2 | 60.1 | 60.7 | 60.8 | 60.3 | 44.1 | 43.1 | 40.4 | 40.8 |
| SH-C1 | 69.9 | 69.9 | 70.1 | 70.2 | 60.6 | 60.7 | 60.7 | 60.1 | 43.3 | 42.4 | 39.9 | 40.3 |
| SH-C2 | 70.1 | 70.2 | 70.2 | 69.9 | 61.2 | 61.6 | 61.5 | 61.1 | 43.3 | 41.6 | 40.5 | 40.4 |
| SH-C3 | 70.2 | 70.4 | 70.3 | 70.1 | 61.1 | 61.6 | 61.6 | 61.2 | 43.4 | 41.6 | 40 | 40.1 |
| SH-C4 | 70.2 | 70.4 | 70.3 | 70.1 | 61.6 | 62 | 61.8 | 61.1 | 43 | 41.5 | 40.5 | 41 |
| SH-C5 | 69.9 | 70.1 | 70.2 | 70.2 | 60.7 | 61 | 60.8 | 60.1 | 42.9 | 41.8 | 39.7 | 40.3 |
| SH-C6 | 70.2 | 70.3 | 70.3 | 70.1 | 61 | 61.5 | 61.6 | 61.1 | 43.6 | 41.8 | 39.9 | 40 |
| SH-C7 | 70.1 | 70.2 | 70.2 | 70.1 | 61.4 | 61.8 | 61.7 | 61.1 | 43.6 | 42 | 40.7 | 41.1 |
| SH-C8 | 69.9 | 70.1 | 70.2 | 70.2 | 60.4 | 60.3 | 60.7 | 60.2 | 43.1 | 42.2 | 39.2 | 39.4 |
| SH-C9 | 70 | 70.1 | 70.2 | 69.9 | 61.1 | 61.5 | 61.5 | 61 | 43.4 | 41.9 | 40.3 | 40.4 |

Note: Four TTMV strains are TTMV-CBD279 (AB290918), TTMV-CBD231 (AB026930), TTMV-CLC138 (AB038626) and TTMV-CLC205 (AB038628).
