# Supplementary material for: Identification of a Torque Teno Mini Virus (TTMV) in Hodgkin’s Lymphoma Patients
Source: Front Microbiol. 2018 Jul 26;9:1680. doi: 10.3389/fmicb.2018.01680 (PMC6070622; doi:10.3389/fmicb.2018.01680)
Supplement: Supplementary file 10 [file Table_7.DOCX]

**Supplementary table 7. Genome and ORFs sequence similarity of TTMV isolate SH-C4 with reference TTMV strains**

| **TTMV strain (GenBank accession NO.)** | **Nucleotide, %** | | | | **Amino acid, %** | | |
| --- | --- | --- | --- | --- | --- | --- | --- |
|  | **Genome** | **ORF1** | **ORF2** | **ORF3** | **ORF1** | **ORF2** | **ORF3** |
| TLMV-CBD279 (AB026931) | 70.2 | 61.6 | 63.1 | 60 | 57.3 | 58.3 | 54.8 |
| TLMV-CBD231 (AB026930) | 70.4 | 62 | 62 | 59.4 | 58.1 | 55.6 | 54.8 |
| TLMV-CLC138 (AB038626) | 70.3 | 61.8 | 59.6 | 60 | 60 | 51.7 | 56.5 |
| TLMV-CLC205 (AB038628) | 70.1 | 61.1 | 58.3 | 59.8 | 59.4 | 51.7 | 56.1 |
| TLMV-NLC023 (AB038629) | 54.8 | 51.3 | 44.6 | 56.1 | 48.3 | 35.1 | 51.5 |
| TLMV-NLC026 (AB038630) | 57 | 57.1 | 41.9 | 56.8 | 46.4 | 31.1 | 53.6 |
| TTMV_Pt-TTV8 (AB041963) | 54 | 58.2 | 47.2 | 73.2 | 45.7 | 37.1 | 73.2 |
| TTMV-TGP96 (AB041962) | 54.4 | 51.1 | 40.4 | 61.9 | 37.3 | 31.8 | 59.4 |
| TTMV_LY2 (JX134045) | 56.4 | 51.9 | 40.8 | 62.2 | 39.1 | 31.8 | 57.3 |
| TLMV-CBD203 (AB026929) | 55.5 | 53.9 | 40.2 | 54.1 | 43.3 | 31.1 | 47.3 |
| TTMV-LIL-y4 (EF538883) | 54.6 | 51.9 | 39.1 | 73.2 | 42.8 | 29.8 | 73.2 |
| TLMV-CLC156 (AB038627) | 55.7 | 55.8 | 35.1 | 49.2 | 41.4 | 25.8 | 43.5 |
| TTMV-PB4TL (AF291073) | 55 | 55.1 | 39.3 | 73.2 | 42.1 | 29.1 | 73.2 |
| TLMV-NLC030 (AB038631) | 54 | 53.6 | 38.4 | 62.1 | 40.4 | 27.8 | 58.6 |
| TLMV-CLC062 (AB038625) | 54.1 | 53.7 | 38.9 | 62.1 | 40.6 | 28.5 | 58.6 |
| patent_TTMV9 (JC018689_) | 54 | 53.8 | 43.7 | 62.1 | 40.7 | 28.5 | 58.6 |
| TTMV-LIL-y1 (EF538880) | 55.3 | 50.5 | 38.6 | 65.3 | 42.2 | 29.1 | 59.8 |
| TTMV-LIL-y2 (EF538881) | 53.7 | 53.4 | 41.7 | 30.4 | 40.6 | 31.1 | 25.9 |
| TTMV-LIL-y3 (EF538882) | 53.5 | 48.2 | 35.1 | 63.7 | 40.6 | 25.2 | 57.3 |
| TTMV_LY1 (JX134044) | 55 | 55.8 | 35.8 | 69.2 | 41.2 | 28.5 | 65.7 |
| TTMV_LY3 (JX134046) | 54 | 51.9 | 34.2 | 56.9 | 40 | 24.5 | 51.9 |
| TTMV-D11 (KF764701) | 54.1 | 53.3 | 32.2 | 55.8 | 41.7 | 24.5 | 48.5 |
| TTMV-D50 (KF764702) | 54.2 | 53.5 | 32.2 | 55.8 | 41.7 | 24.5 | 48.5 |
| TTMV-ALA22 (KM259873) | 55.5 | 46.5 | 39.1 | 56.1 | 39.2 | 26.5 | 51.9 |
| TTMV-ALH8 (KM259874) | 55.9 | 50.1 | 34.4 | 60.8 | 42.7 | 25.8 | 57.3 |
| TTMV-222 (KU041847) | 64.7 | 58.6 | 50.1 | 62.1 | 54.4 | 40.4 | 58.2 |
| TTMV-Emory1 (KX810063) | 53.8 | 50.7 | 37.3 | 64.4 | 41.7 | 32.5 | 59.4 |
| TTMV-Emory2 (KX810064) | 54.1 | 53 | 36 | 77.7 | 42.7 | 28.5 | 72.8 |
