# Supplementary material for: Identification of a Torque Teno Mini Virus (TTMV) in Hodgkin’s Lymphoma Patients
Source: Front Microbiol. 2018 Jul 26;9:1680. doi: 10.3389/fmicb.2018.01680 (PMC6070622; doi:10.3389/fmicb.2018.01680)
Supplement: Supplementary file 11 [file Table_8.DOCX]

**Supplementary table 8. Relationship between Hodgkin’s lymphoma and HIV, HTLV and EB virus**

| **Virus** | **Group** | **n*** | **Total** | **% with virus** | ***P***** |
| --- | --- | --- | --- | --- | --- |
| **HIV** | **Hodgkin’s lymphoma** |  |  |  |  |
|  | Positive | 0 | 19 | 0 | >0.5 |
|  | Negative | 5 | 292 | 1.7 |  |
|  |  |  |  |  |  |
|  | Hodgkin's lymphoma positive | 0 | 19 | 0 | / |
|  | Health | 0 | 40 | 0 |  |
| **HTLV** | **Hodgkin’s lymphoma** |  |  |  |  |
|  | Positive | 0 | 19 | 0 | >0.5 |
|  | Negative | 2 | 292 | 0.7 |  |
|  |  |  |  |  |  |
|  | Hodgkin's lymphoma positive | 0 | 19 | 0 | / |
|  | Health | 0 | 40 | 0 |  |
| **EB virus** | **Hodgkin’s lymphoma** |  |  |  |  |
|  | Positive | 0 | 19 | 0 | >0.5 |
|  | Negative | 1 | 292 | 0.3 |  |
|  |  |  |  |  |  |
|  | Hodgkin's lymphoma positive | 0 | 19 | 0 | / |
|  | Health | 0 | 40 | 0 |  |

*: “n” means virus positive sample number.

**: *P* value was made using Fisher’s exact test.
